# The plastoquinone pool, poised for cyclic electron flow?

**DOI:** 10.3389/fpls.2015.00540

**Published:** 2015-07-28

**Authors:** Jean Alric

**Affiliations:** Unité Mixte de Recherche 7265 Biologie Végétale et Microbiologie Environnementales, Laboratoire de Bioénergétique et de Biotechnologie des Bactéries et des Microalgues, Centre National de la Recherche Scientifique, Commissariat à l' Energie Atomique, Institut de Biologie Environmentale et BiotechnologieSaint-Paul-lez-Durance, France

**Keywords:** PGR5, NDH, PGRL1, *Chlamydomonas reinhardtii*, cytochrome *b*_6_*f* complex

The relative contribution of cyclic compared to linear electron flow (LEF) depends on the redox poise of electron carriers, and is also dependent upon the relative turnovers of the two photosystems (only photosystem I is involved in cyclic). Although these views have been built on solid experimental facts gathered over the years ~1963–1987, they have sometimes been overlooked in the recent literature. In the microalga *Chlamydomonas reinhardtii*, a PSI-cyt *b*_6_*f* supercomplex is formed when cells are incubated under anaerobic conditions and PSI antenna size is large (state 2), conditions that are thought to be favorable to cyclic electron flow (CEF). Although a more reductive poise of the chloroplast and an increase in PSI antenna size would expectedly speed up cyclic turnovers in limiting light, the role for supercomplex formation in favoring cyclic flow has not yet been fully demonstrated. Linear and cyclic are indeed concurrent and competing flows, however, evidence is still lacking that the sequestration of PSI inside a cyclic supercomplex decreases linear flow.

## Introduction

Electron and proton transfer plays a central role in coupling light capture to the chemical reactions of carbon metabolism. Electrons, originating from charge separation in the photosystems, are eventually transferred to NADP^+^ to form NADPH; it is denoted as linear electron flow (LEF). Protons, coupled to electrons for neutral diffusion through the thylakoid membrane, are translocated by ATPase for the synthesis of ATP. Carbon metabolism has to keep pace with light capture and it relies on an electron transfer feedback loop: cyclic electron flow (CEF) around PSI (Whatley et al., 1955). In C3 photosynthesis, an upstream limitation bears at the level of rubisco under limiting CO_2_ (Farquhar et al., 1980). Electrons that cannot be extracted from NADPH to form G3P are recycled around cytochrome *b*_6_*f* and PSI, contributing to the proton motive force, pmf. This pmf is used for ATP synthesis and the regeneration of RuBP from G3P with limitation bearing at the level of SBPase, cytochrome *b*_6_*f* complex and ATPase under non-limiting CO_2_ (Farquhar et al., 1980; Raines, 2003; Yamori et al., 2011). The lifetime of excited chlorophyll is also decreased by the ΔpH component of pmf [qE component of non-photochemical quenching (Gilmore and Björkman, 1995)], thereby limiting the reducing pressure of the photosystems. In high light, nonetheless, electrons accumulate in the plastoquinone pool, favoring an increase of PSI antenna size [state transitions (Allen et al., 1981)]. The oxidized form of PSI primary electron donor P^+^_700_ can accumulate in the light, showing that intersystem electron transfer is limited upstream at the level of cytochrome *b*_6_*f* activity. This latter limitation, also denoted as “photosynthetic control,” is pH-dependent (Kok et al., 1969) and induced by CEF (Johnson et al., 2014b) thereby providing a feed-back down-regulation of linear electron transfer. The proton motive force equilibrates with the ATP/ADP ratio, as a function of the ATPase coupling factor. The intermeshed nature of these reactions defy our understanding of the photosynthetic process as a whole, and represents a challenge for future redesigns of photosynthesis, aiming at minimizing wasteful reactions, whether it is at the level of light capture, electron/proton transfer or carbon metabolism.

## Basic kinetics and thermodynamics of CEF

### The kinetically limiting step in CEF is the reduction of plastoquinone

It has been established that the limiting step for CEF is the reduction of plastoquinone. Maxwell and Biggins wrote ≪ the rate-limiting step for this pathway must reside between P^−^_430_ and plastoquinone ≫ (Maxwell and Biggins, 1976), where “P^−^_430_” referred to the reduced electron acceptors of PSI. This conclusion was drawn because the P^+^_700_ reduction rate is much slower in the presence of DCMU (CEF only) than it is in its absence (mostly LEF), see also (Alric et al., 2010). It shows that cytochrome *b*_6_*f* is not limiting for CEF.

### Partial inhibition of PSII increases CEF

It has been shown quite consistently that the photochemical action of PSII, decreasing the concentration of oxidized quinones, diminishes the rate of CEF. It is generally accepted that LEF and CEF are in competition with each other. There is an optimal amount of active PSII for a proper redox poise of the chain to sustain CEF (e.g., an optimal non-saturating DCMU concentration, see Tagawa et al., 1963); below this optimal concentration in active PSII, the concentration in reduced ferredoxin may become limiting to reduce plastoquinones; beyond this point oxidized plastoquinones may be lacking to accept electrons from ferredoxin. The conspicuous role of PSII in providing a proper redox poise for CEF has been confirmed in a number of subsequent studies (Avron and Neumann, 1968; Arnon and Chain, 1975; Mills et al., 1978; Slovacek et al., 1979; Hosler and Yocum, 1987; Bendall and Manasse, 1995; Allen, 2003).

### Role of state transitions under limiting light

The use of monochromatic actinic light in the spectral region around 700 nm shows that, concurrent to the “red drop” of LEF, a “red rise” of CEF is observed (Arnon et al., 1967). It again demonstrates that CEF is stimulated when PSI excitation is favored at the expense of PSII. Another way to rebalance light excitation between the two photosystems is to play with state transitions. Under limiting light conditions, State 2 will at first favor the excitation of PSI compared to PSII, and would therefore be expected to promote CEF at the expense of LEF. This initial imbalance is only transitory because state transitions are essentially a reversible process: they constantly readjust photosystems antenna sizes as a function of the redox poise of the PQ pool (Allen et al., 1981). Therefore, under steady state, this reversible process favors neither CEF at the expense of LEF nor the otherwise, it just poises the electron input from the two photosystems so that LEF and CEF are in equilibrium. Under light conditions that could be considered saturating, the Chlamydomonas *stt7* mutant, blocked in State 1, shows normal CEF (Lucker and Kramer, 2013) especially when DCMU is present (Takahashi et al., 2013; Alric, 2014), suggesting that CEF is redox-dependent rather than controlled by state transitions in some mechanical kind of a way.

## Lateral heterogeneity in photosynthetic membranes and possible consequences on CEF in high light, a general perspective

### Heterogeneities and restricted microdomains for quinone diffusion

PSII is mostly localized in the appressed regions of thylakoids (grana stacks) whereas PSI localizes preferentially to non-appressed regions (grana margins and stroma lamellae) (Andersson and Anderson, 1980). Cytochrome *b*_6_*f* complex is equally spread in the stacked and unstacked regions (Cox and Andersson, 1981; Anderson, 1982), suggesting that plastocyanin (and not plastoquinone) serves as the long-distance electron carrier between PSII and PSI. NDH (Berger et al., 1993; Lennon et al., 2003) and PGRL1 (Hertle et al., 2013) localize, together with PSI, in the grana margins and stroma lamellae. Such co-localization to the thylakoid regions exposed to the stroma makes sense because PSI as well as NDH and PGR have to interact with soluble electron carriers diffusing in the stroma: ferredoxin, FNR and NADP^+^/NADPH. The experiments of Joliot and Lavergne support plastocyanin (and not plastoquinone) as the long-distance electron carrier between PSII and PSI (Joliot et al., 1992; Lavergne et al., 1992). Their work showed the compartmentalization of PSII and plastoquinones in heterogeneous membrane micro-domains containing on average about 3–4 PSII centers and about 6 PQs per PSII center. This “local pool” of plastoquinones around PSII proved to be rapidly photoreduced (< 100 ms) whereas equilibration with the whole PQ pool occured in the range of a few seconds. On the other hand, only two thirds of cyt *f* is rapidly rereduced from PQH_2_ formed by PSII in the grana stacks (Joliot and Joliot, 1992), suggesting that the other third of cyt *f* is disconnected from PSII and probably corresponds to the fraction found in the lamellae.

### Consequences for CEF in saturating light

If the thylakoid membranes were a homogenous entity, PSII would almost fully reduce the PQ pool under conditions of saturating light and therefore LEF would significantly oppose CEF. Some of the photosynthetic apparatus must be operating like this because otherwise the addition of non-saturating concentrations of DCMU would not stimulate CEF (see above); but on the other hand, NPQ measurements done in the absence of DCMU suggest that CEF remains significantly active in saturating light. It has been extensively reported that under light intensities >1000 μmols.m^−2^.s^−1^ the ΔpH-dependent (nigericin-sensitive) qE component of non-photochemical quenching of chlorophyll fluorescence is severely decreased in *pgr5* and *pgrl1* mutants, in Arabidopsis (Munekage et al., 2002; Dalcorso et al., 2008; Joliot and Alric, 2013) as well as in Chlamydomonas (Peers et al., 2009; Tolleter et al., 2011; Johnson et al., 2014b). Such a prominent role for CEF in very high light would not be expected if the system were completely homogenous. Under saturating illumination where the photochemical rate of PQ reduction largely exceeds non-photochemical PQ reduction by the NDH or PGR pathways, the persistence of CEF can then be attributed, for a good part, to the lateral heterogeneity of the thylakoid membrane: the PQ pool may not be homogenously reduced by PSII, some oxidized PQs may be retained locally in the grana margins and the stroma lamellae from which PSII is excluded. Oxidized plastoquinones are electron acceptors for NDH or PGR, localized in the non-appressed regions of thylakoids. The localization of cyt *b*_6_*f* complex in these regions might be indispensable to the regeneration of oxidized plastoquinones. The enrichment of unstacked membranes in cyt *b*_6_*f* complexes upon transition to State 2 (Vallon et al., 1991) does not translate into a significant increase in the measured CEF rate (Takahashi et al., 2013; Alric, 2014). It simply shows again that cyt *b*_6_*f* complex is not the limiting step for CEF.

### A functional role for supercomplexes?

The presence of NADPH dehydrogenases and PGR5/PGRL1 proteins in both higher plants and green algae suggests that the mechanism for CEF must be conserved throughout the green lineage despite the differences that may exist between photosynthetic organisms (Peltier et al., 2010). Nevertheless, green algae often show little stacking of the thylakoid membranes whereas state transitions seem more pronounced in green algae than in higher plants (Johnson et al., 2014a). PSI and cyt *b*_6_*f* co-purify in Chlamydomonas cells acclimated to state 2 conditions (Wollman and Bulte, 1989; Iwai et al., 2010), but no similar association was found in spinach (Breyton et al., 2006). In a more evolutionary context, the structural diversity observed in different species may require different strategies for segregation of plastoquinones—which is why some have predicted a need for supercomplexes in green algae (Johnson et al., 2014a). In contrast, higher plant thylakoid stacking changes dynamically in response to light (Rozak et al., 2002) and CEF accelerates with increasing light intensity, even after saturation of LEF (Kou et al., 2013). It suggests that the thylakoid membrane may not only represent a static frame where photosynthetic complexes are embedded, but that lateral heterogeneities and membrane dynamics may also act as a control point for regulating photosynthesis.

Although these structural differences are easily pictured, it is very difficult to address the question of how they impact the kinetics of photosynthetic electron flow. In a recent book chapter summarizing most of his kinetics studies on restricted diffusion of quinones in membrane microdomains, Lavergne wrote (Lavergne, 2009) p. 203 ≪ The problem is that very different organizations can have similar effects […], the “low apparent equilibrium constant” is a good diagnostic for non-homogeneity, but it does not suffice to distinguish between a “crystalline,” ordered arrangement (e.g., the supercomplex model), a distributed stoichiometry due to small size confinement […], or large scale stoichiometric heterogeneity (as probably occurs in thylakoids). ≫ So the question remains as to whether or not the association of PSI and cyt *b*_6_*f* in supercomplexes favor CEF more than the exclusion of PSII from the stroma lamellae does, if it has any effect at all. The recent isolation of mutants such as *curt* that have altered thylakoid structure illustrates the importance of lateral heterogeneity for an optimal photosynthesis (Armbruster et al., 2013). Such mutants modified in their granal structure or others impaired for supercomplex formation will contribute to future investigations and contribute to the ongoing debate around this subject.

### Conflict of interest statement

The author declares that the research was conducted in the absence of any commercial or financial relationships that could be construed as a potential conflict of interest.
